# Virulence and pathogenicity determinants in whole genome sequence of *Fusarium udum* causing wilt of pigeon pea

**DOI:** 10.3389/fmicb.2023.1066096

**Published:** 2023-02-17

**Authors:** Alok K. Srivastava, Ruchi Srivastava, Jagriti Yadav, Alok K. Singh, Praveen K. Tiwari, Anchal K. Srivastava, Pramod K. Sahu, Shiv M. Singh, Prem Lal Kashyap

**Affiliations:** ^1^ICAR-National Bureau of Agriculturally Important Microorganisms (NBAIM), Maunath Bhanjan, Uttar Pradesh, India; ^2^Centre of Advanced Study in Botany, Institute of Science, Banaras Hindu University, Varanasi, Uttar Pradesh, India; ^3^ICAR-Indian Institute of Wheat and Barley Research, Karnal, Haryana, India

**Keywords:** *Fusarium udum*, wilt, whole genome sequence, virulence determinants, effector proteins, *SIX* genes pathogenicity determinants in Fusarium udum genome

## Abstract

The present study deals with whole genome analysis of *Fusarium udum*, a wilt causing pathogen of pigeon pea. The *de novo* assembly identified a total of 16,179 protein-coding genes, of which 11,892 genes (73.50%) were annotated using BlastP and 8,928 genes (55.18%) from KOG annotation. In addition, 5,134 unique InterPro domains were detected in the annotated genes. Apart from this, we also analyzed genome sequence for key pathogenic genes involved in virulence, and identified 1,060 genes (6.55%) as virulence genes as per the PHI-BASE database. The secretome profiling of these virulence genes indicated the presence of 1,439 secretory proteins. Of those, an annotation of 506 predicted secretory proteins through CAZyme database indicated maximum abundance of Glycosyl hydrolase (GH, 45%) family proteins followed by auxiliary activity (AA) family proteins. Interestingly, the presence of effectors for cell wall degradation, pectin degradation, and host cell death was found. The genome comprised approximately 895,132 bp of repetitive elements, which includes 128 long terminal repeats (LTRs), and 4,921 simple sequence repeats (SSRs) of 80,875 bp length. The comparative mining of effector genes among different *Fusarium* species revealed five common and two specific effectors in *F. udum* that are related to host cell death. Furthermore, wet lab experiment validated the presence of effector genes like *SIX* (for Secreted in Xylem). We conclude that deciphering the whole genome of *F. udum* would be instrumental in understanding evolution, virulence determinants, host-pathogen interaction, possible control strategies, ecological behavior, and many other complexities of the pathogen.

## Introduction

The challenge of increasing food grain production is rising day by day as the global population continues to rise. It is projected to increase by another 3.5 billion people before the end of this century, reaching an estimated 11.2 billion (Roser, 2019). Pigeon pea (*Cajanus cajan* L. Millsp.) is an economically significant grain legume crop of Fabaceae family native to semi-arid tropics of the world, such as India, Bangladesh, Indonesia, Thailand, Mauritius, Kenya, Ghana, Tanzania, Uganda, Malawi, and Trinidad (Singh et al., 2016). The crop was the fifth-ranked pulse crop worldwide, accounting for 91% of global production. Pigeon pea has an average yield of 0.97 t ha^−1^ and is cultivated globally on 7.02 million hectares of land in Asia, Africa, and Latin America (FAOSTAT, 2017). Legumes, preferably pigeon pea, is an acceptable substitute for readily available protein sources. It provides a significant amount of food protein in the Indian and African subcontinents, while requiring little in the way of cultivation care and inputs. This crop makes a significant contribution by meeting about 20% of the world population’s protein needs and also serves as a substantial source of other nutrients. In India, pigeon pea stand in the second position after chickpea (Allen and Lenné, 1998). Among the various biotic stresses, vascular wilt caused by *Fusarium udum* is one of the most devastating disease in pigeon pea. It was reported to pose annual output losses of about 71 million USD in India (Reddy et al., 2012; Kashyap et al., 2015). It was later reported from other countries belonging to South Asia, Africa, and Europe (Nene and Reddy, 1976). Pigeon pea wilt can cause yield losses of up to 67% at maturity and 100% in cases of infection at the pre-pod stage (Kannaiyan and Nene, 1981). Additionally, Mahesh et al. (2010), Karimi et al. (2012), Pawar et al. (2013), Prasad et al. (2012), and Kumar and Upadhyay (2014), found comparable findings of pigeon pea wilt disease.

*Fusarium udum* is a soil-borne fungus with no sexual stage known. The asexual spores are of three types: microconidia, macroconidia, and chlamydospores. Micro-conidia are regularly produced under all conditions (Purohit et al., 2017). Macroconidia is a 2–6-celled sporodochia-like structure produced on the host surface. Similarly, the chlamydospores are produced in the older mycelium. Among these three asexual spores, chlamydospores survive for a considerably long time in the soil. This is an important source of inoculum for the next crop. *F. udum* causes wilting at the flowering stage and symptoms can also be seen at the seedling stage (Choudhary, 2010). Once this pathogen gets established in the vascular bundle, the mycelium, spores, and polysaccharides get filled into the xylem vessels. The water and nutrient transport in the xylem is further reduced by shortening the xylem parenchyma cells due to stimulated cell division by fungus (Agrios, 2008). Pathogen-emitted toxins are conveyed to the leaves, diminishing chlorophyll amalgamation and penetrability of the leaf cell layers and their capacity to control water misfortune through transpiration, consequently causing wilting, interveinal necrosis, yellowing, and plant death. Breeding for resistance to pigeon pea wilt mainly relies on the genetic variability present among different *F. udum* strains. Detailed information on morphological and pathogenic diversity is available (Kashyap et al., 2015), but physiological diversity at a molecular level is yet to be explored.

Whole genome sequencing (WGS) data generated from this study is currently available in the public database (Srivastava et al., 2018). *De novo* draft genome sequencing and functional annotation of *F. udum* have been done to understand the molecular function. The advancement of next-generation sequencing (NGS) technologies and the decrease in the price of sequencing for each sample have undoubtedly accelerated the process of determining emerging genes and genomic regions (Schneeberger et al., 2009). As a result, many current methods utilizing Bulked-Segregant Analysis (BSA) combined with whole-genome re-sequencing (WGRS) for rapid identification of specific genes of interest in plants called “quick forward genetics” (Mokry et al., 2011). For instance, in the model crop *Arabidopsis*, which has a genome size of about 135 Mb, was successfully used to test NGS based BSA approaches for the identification of potential genes for leaf color, next-generation mapping, and suppressor mutants (Schneeberger et al., 2009). In case of plant-pathogen interaction, genome sequencing can reveal virulence-related genes for a better understanding of host-pathogen communications. Currently, biological and chemical disease management approaches adopted for the management of wilt fungus are not successful. So far, the genes responsible for pathogenicity of *F. udum* has not yet been investigated at molecular level (Uchida et al., 2011). Therefore, through this study we performed whole genome analysis of most virulent strain of *F. udum* and identified putative virulence genes for understanding molecular basis of host-pathogen interactions in pigeon pea. The information generated from this study will be highly useful in resistance breeding against *Fusarium* wilt of pigeon pea.

## Materials and methods

### Pathogenicity test and selection of virulent strain

Total seven strains of *F. udum* (F-02842, F-02843, F-02844, F-02845, F-02848, F-02850 and F-02851) were collected from NAIMCC (ICAR-NBAIM) for screening and identification of the most virulent strain (Supplementary Table S1). The disease-causing ability of these strains were tested by following the protocol of Nene et al. (1981). All the strains were grown separately in potato dextrose broth and incubated at 28°C for 2–7 days. Prepared sand pigeon pea flour medium and inoculated with selected cultures of *F. udum* and incubated at room temperature for 15 days. The 200 g of fungus-infested sand pigeon pea flour medium was mixed with 2 kg autoclaved soil and the mixture was placed into the pot and kept for 2 days. After 2 days, the cell count (6 × 10^5^ spore mL^−1^) was optimized. In the pot experiment, four varieties of pigeon pea seeds were sown: BAHAR, BRG2, DA 11, and MAL 11. Surface sterilization of seeds was performed with 0.5% (v/v) NaOCl for 10 min, followed by washing with deionized water thrice (Sahu et al., 2022). For screening, 10 seeds of each varieties were sown separately in each pots. Plants were raised with standard agronomic practices. Wilt was observed in pigeon pea plants up to 60 days and permanent wilting of plants were recorded in each treatments.

### Comparative gene analysis

The whole genome sequencing of most virulent strain *F. udum* F-02845 was performed and submitted to NCBI database (Srivastava et al., 2018). After retrieving genome sequence, a comparative analysis of orthologous gene families was carried out across *Fusarium* species. The orthologous groups among *F. udum* F-02845, *F. acutatum* (JAADJF000000000), *F. graminearum* PH 1, *F. mangiferae* (FCQH00000000), *F. oxysporum* 4,287, and *F. fujikuroi* IMI 58289 were identified using the OrthoVenn2 program (Xu et al., 2019) with a threshold E value ≤1e−5 and an inflation value 1.5. A workflow followed for the analysis followed in the present study is given in the supplementary data (Supplementary Figure S1).

Since there was one more genome of *F. udum* was reported during the analysis (*F. udum* NRL 25194), the predicted proteome of *F. udum* F-02845 was compared with the *F. udum* NRL 25194 using Orthovenn2.

### Repetitive sequence analysis

RepeatMasker (v4.0.5) was used to screen the nucleotide sequences for low complexity DNA sequences and the interspersed repeat (ISR) was used to identify transposable DNA elements. Microsatellite repeats were identified in sequence using the MISA Pearl script tool^1^ (Beier et al., 2017), and results were further authenticated on the WEBSAT server^2^ (Martins et al., 2009).

### Gene prediction

On the basis of a database of eukaryotic genomes, GeneMark-ES and AUGUSTUS were used to predict the genes in the *F. udum* genome. With default parameters, the above programes are highly reliable for accurate gene prediction^3^ (Stanke et al., 2004).

### Functional annotation and pathway enrichment analysis

The BLASTx homology search tool, a component of the standalone NCBI-blast-2.3.0+, was used to perform functional annotation of the *F. udum* genes (Altschul et al., 1990). With a cut-off E value of ≤1e−06 and a similarity of 34%, BLASTx identified the homologous sequences of the genes in the NCBI non-redundant protein database. Gene ontology (GO) analysis was carried out using Blast2GO PRO 4.1.5 (Conesa and Gotz, 2008). In three different mappings, B2G performed as follows: (1) Using two NCBI-provided mapping files, blast result accessions are used to get gene names (symbols; gene info, gene 2 accessions). (2) Blast result GI identifiers were used to retrieve UniProt IDs using a mapping file from PIR (non-redundant reference protein database), which includes PSD, Swiss-Prot, UniProt, TrEMBL, GenPept, RefSeq, and PDB. The names of the identified genes were searched in the species-specific entries of the gene product table of the GO database. With the aid of the KAAS-KEGG Automatic Annotation Server, pathway analyses were carried out. This database provides functional annotation of genes using other data servers (Moriya et al., 2007). Accessions from the blast results were looked for in the DBXRef table of the GO database.

### *In silico* mining of virulence genes

A web-based database called the Pathogen-Host Interaction Database (PHI-base; Winnenburg et al., 2006), which comprises experimentally verified pathogenicity, virulence, and effector genes from bacterial and fungal pathogens that infect hosts like plants, animals, fungi, and insects. BLASTP was employed with a cut-off E value of ≤1e−06 in the pathogen-host interaction (PHI) database to find the probable pathogenicity-related genes.

### Secretome prediction and analysis of secretory effectors

In order to determine the secretory signal peptides, SignalP v4.1 (Nielsen, 2017)^4^ was used to examine the 16,179 predicted proteins of *F. udum*. Further, TMHMM v2.0 was used to predict the protein sets with the existence of transmembrane domains (Krogh et al., 2001) and GPI (glycosylphosphatidyl inositol)-anchor using PredGPI (Pierleoni et al., 2008). Proteins including one transmembrane domain situated within the N-terminal signal peptide and no transmembrane domain overall were chosen. The predicted secretory proteins’ cysteine content was examined. In order to functionally annotate the predicted secretome, BLAST2GO was used to assign GO keywords (Altschul et al., 1990). The dbCAN HMMs 5.0 (Yin et al., 2012) was used to find carbohydrate metabolism active enzymes (CAZymes) based on the CAZy database in the *F. udum* secretome.

### PCR validation of effector genes

In order to validate the annotated genes, we designed 14 genic-SSRs targeting *SIX* family genes of different *F. udum* strains (F-02842, F-02843, F-02844, F-02845, F-02848, F-02850 and F02851). For PCR amplification of 10 μl reaction volume constituting of template DNA (50 ng μl^−1^), Go Taq Green master mix (Promega Biotech India Pvt. Ltd) and primers (both reverse and forward) was used. Amplification was performed by following conditions of 35 cycles of 95°C for 1 min, primer annealing variable for each primer, and extension at 72°C for 2 min, followed by a hold at 72°C for 5 min. The experiment was repeated three times to confirm the results.

## Results

### Pathogenicity test

Out of seven strains used for pathogenicity test, strain *F. udum* F-02845 showed highest disease incidence in the BAHAR variety, suggested most virulent of all seven strains of *F. udum.* While, F-0244 had least disease incidence in pigeon pea plants (Supplementary Table S1; Supplementary Figure S2). Pigeon pea plants which were showing the highest disease incidence were taken for re-isolation of *F. udum* on PDA plates and DNA was isolated by the CTAB method (Kaul et al., 2022) and quantified by nanodrop. The most virulent strain F-02845 was chosen for whole genome sequencing.

### Gene prediction and functional annotation

**Figure 1:**
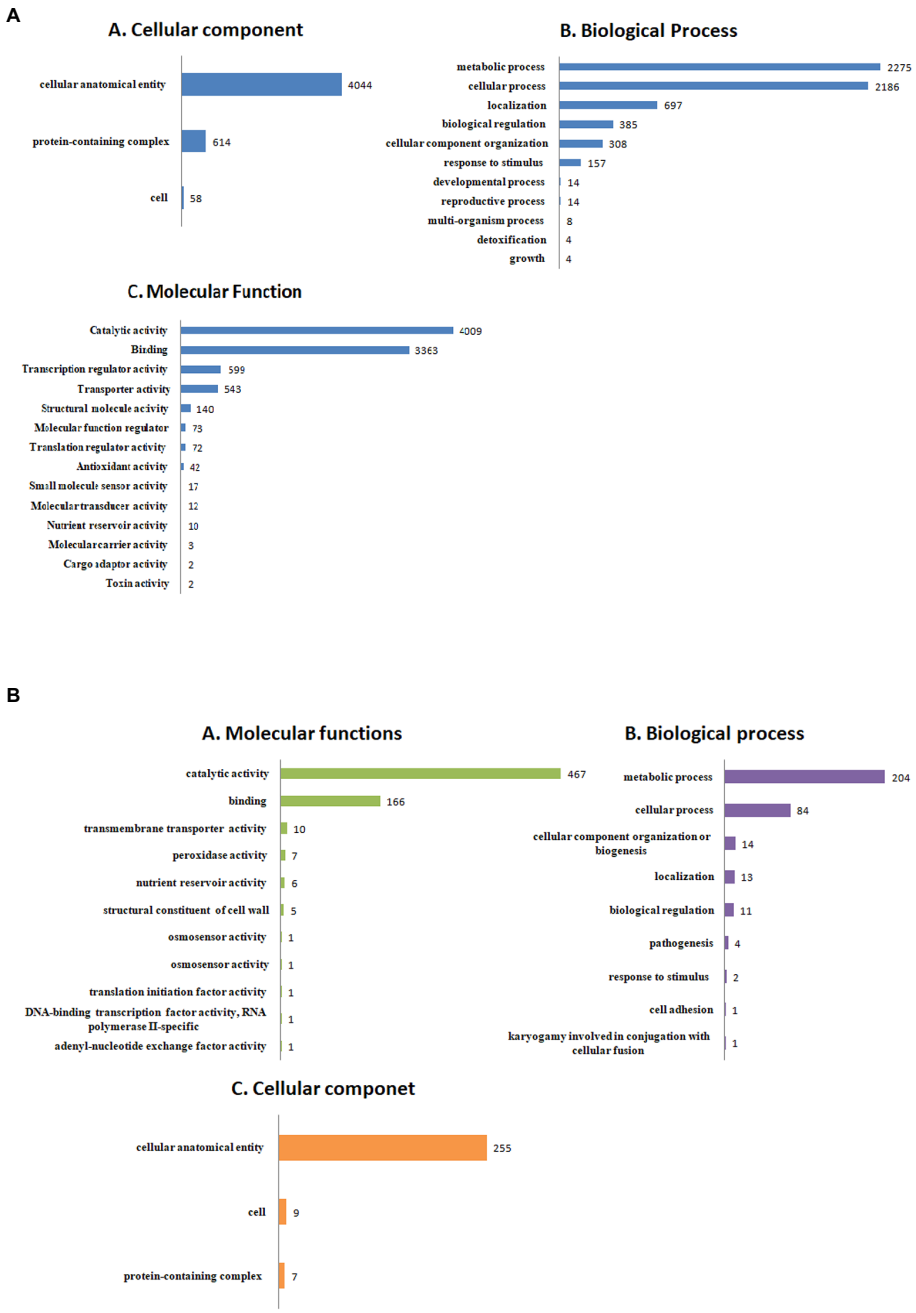
Functional annotation of the *Fusarium udum* **(A)** proteome, and **(B)** secretome showing top hits of different categories.

The detailed information about the genome assembly statistics is provided in Supplementary Table S2. Functional annotation of *F. udum* F-02845 resulted in the identification of 296 tRNAs and 53 rRNAs. A total of 14,673 and 16,179 genes were predicted by using AUGUSTUS and GeneMarkES softwares, respectively. The estimated average length of protein-coding genes was 1,365 bp. Out of 16,179 protein-coding genes, 85.54% of the genes (13,841 genes) were functionally annotated using BlastP (Supplementary Table S3). The gene annotation results indicated that, 3,436 genes belonged to biological processes (BP), with role in metabolic, cellular, localization, biological regulation, cellular component organization, response to stimulus, etc. The 4,511 genes belonged to cellular components (CC) that are part of cellular anatomical entries, protein-containing complexes and cell parts etc. The 6,260 genes belonged to molecular functions (MF) that had catalytic activity, binding, transcription regulatory activity, transporter activity, structural molecule activity, molecular function regulators and antioxidant activity, etc. (Figure 1).

A total of 8,928 genes were categorized into four functional groups using the KOG database. These include: KOG class A (RNA processing and modification, gene count 346), class B (chromatin structure and dynamics, gene count 136), class C (energy production and conversion, gene count 591), and class D (cell cycle control, cell division, chromosome partitioning, and gene count 257; Supplementary Figure S3; Table 1). Further, it has been predicted that the genome contained 1,439 genes encoding for secretory signal peptides, 2,858 for carbohydrate-active enzymes (CAZy), 3,682 for transporter genes, and 1,060 for putative pathogenicity or virulence genes (Table 2). The domain analysis based on InterproScan-V5 revealed the presence of 11,892 annotated genes (73.50%) and 5,134 unique Interpro domains (Supplementary Figure S4).

**Table 1:** Eukaryotic orthologous groups (KOG) classification of the predicted genes within the *Fusarium udum* F-02845 genome.

**#KOG class**	**Count**	**Description**
A	346	RNA processing and modification
B	136	Chromatin structure and dynamics
C	591	Energy production and conversion
D	257	Cell cycle control, cell division, chromosome partitioning
E	530	Amino acid transport and metabolism
F	125	Nucleotide transport and metabolism
G	534	Carbohydrate transport and metabolism
H	141	Coenzyme transport and metabolism
I	537	Lipid transport and metabolism
J	432	Translation, ribosomal structure and biogenesis
K	566	Transcription
L	259	Replication, recombination and repair
M	205	Cell wall/membrane/envelope biogenesis
N	5	Cell motility
O	676	Posttranslational modification, protein turnover, chaperones
P	251	Inorganic ion transport and metabolism
Q	600	Secondary metabolites biosynthesis, transport and catabolism
R	1,615	General function prediction only
S	435	Function unknown
T	789	Signal transduction mechanisms
U	602	Intracellular trafficking, secretion, and vesicular transport
V	114	Defense mechanisms
W	28	Extracellular structures
X	1	multiple functions
Y	40	Nuclear structure
Z	328	Cytoskeleton

Results are grouped into 26 functional classes according to their functions.

**Table 2:** Functional annotation of *Fusarium udum* F-02845 genome.

**Sr. No.**	**NCBI Accession Number**	**NIFK00000000**
1.	Genome size	56.75 Mb
2.	(G + C)%	43.44%
3.	No of gene predicted	16,179
4.	Total no. of genes annotated	13,841
5.	Total no. of repeats identified	1.59%
6.	Total no. of proteins annotated with InterPro domains	11,829
7.	Total no. of genes with gene ontology detected	8,642
8.	No of rRNA identified	53
9.	No. of tRNA identified	296
10.	Total no. of genes annotated with Cluster of Orthologous genes (KOG)	8,928
11.	Total no. of genes coding for carbohydrate active enzymes (CaZy)	2,858
12.	Total no. of pathogenic/virulence genes detected	1,060
13.	Total no. of signal peptide predicted	1,439
14.	Total no. of transmembrane helices predicted	15,649
15.	Total no. of genes coding for transporters	3,682
16.	No.of SSR	4,921

Comparative analysis for orthologus genes families between the predicted proteome of F-02845 and NRL 25194, identified the presence of 13,194 clusters. Out of which, 13,103 common clusters with 26,834 proteins (13,485 of F-02845 and 13,349 of NRL 25194) were shared between F-02845 and NRL 25194. Interestingly, 82 unique clusters with 188 proteins belonged to in F-02845 and 9 unique clusters with 19 proteins belonged to NRL 25194 were identified. There were 2,506 and 7,694 singletons (proteins are not in any clusters) in F-02845 and NRL 25194, respectively, were detected (Supplementary Figures S5, S6; Supplementary Table S4).

### Prediction and analysis of *F. udum* secretome

**Figure 2:**
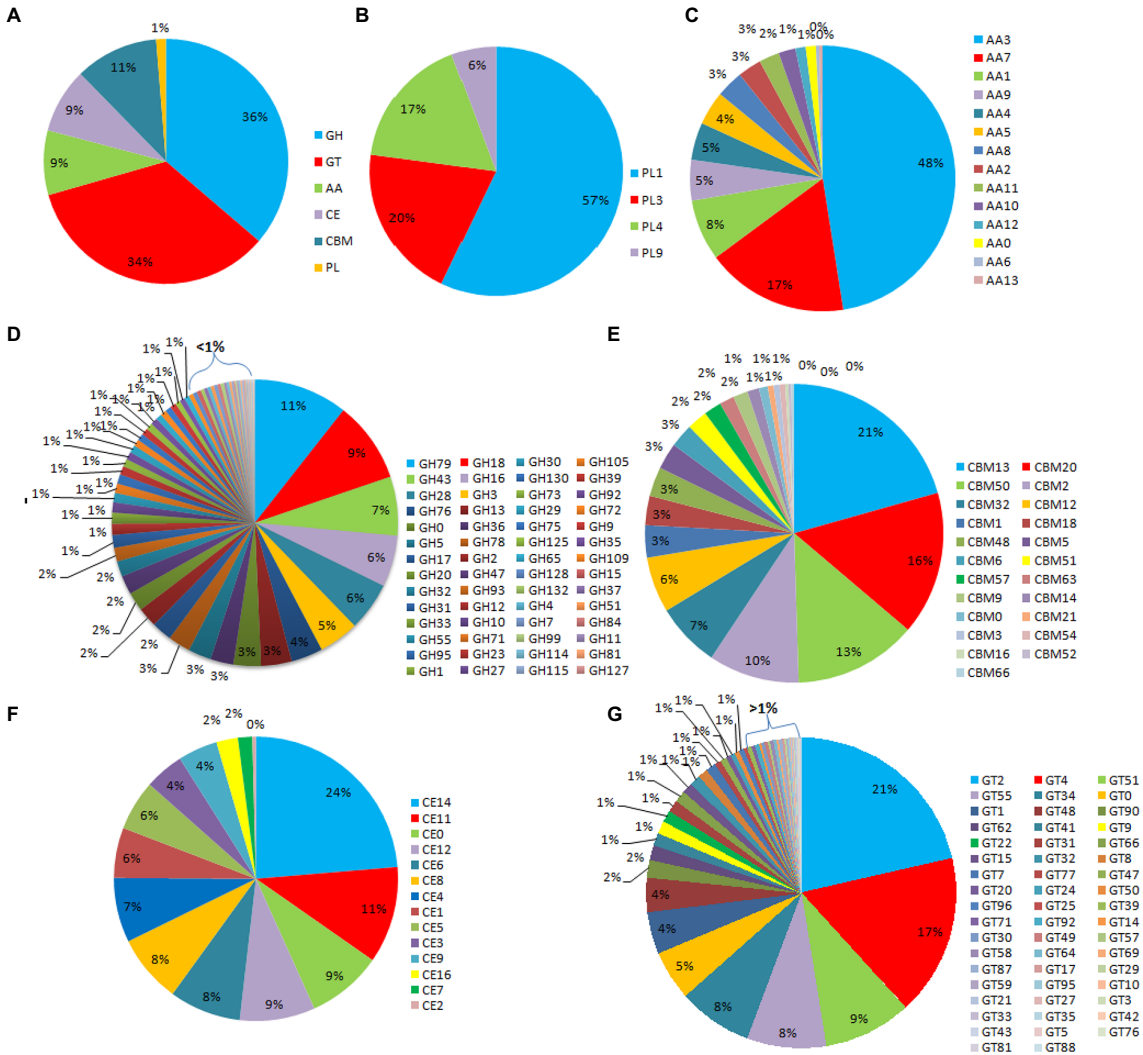
CAZymes identified in the secretome of *Fusarium udum*; **(A)** Summary of the six CAZyme categories: carbohydrate-binding modules (CBMs), carbohydrate esterases (CEs), glycoside hydrolases (GHs), glycosyl transferases (GTs), polysaccharide lyases (PLs), and auxiliary activities (AAs). **(B)** Distinct summaries of the CAZyme PLs, **(C)** Distinct summaries of each of the CAZyme auxiliary activities (AAs), **(D)** Distinct summaries of each of the CAZyme GHs, **(E)** Distinct summaries of each of the CAZyme carbohydrate-binding modules (CBMs), **(F)** Distinct summaries of each of the CAZyme CEs, and **(G)** Distinct summaries of each of the CAZyme GTs.

Out of the 16,179 predicted protein-coding sequences, a total of 1,439 proteins represented classical secretory proteins. Out of 1,439 secretory proteins, 1,305 proteins were functionally annotated and 134 sequences had no hit in the non-redundant database (Supplementary Table S5). A total of 124 highly probable sequences containing GPI anchors were identified and 1,021 proteins had GO terms. Based on the GO, all the genes were divided into several categories that included 240 genes under biological process category, representing, cellular component organization, genesis, localization, and biological regulation. Similarly, under cellular component that included 268 genes that are part of cellular anatomical entities, cells and protein-containing complexes. In molecular function, 513 genes were identified that had catalytic activity, binding, transmembrane transporter activity, peroxidase and nutrient reservoir activity (Figure 1). Additionally, the CAZyme database was used to investigate 1,439 secretory proteins and predicted 506 secretory proteins with plant cell wall degradation functions (Figure 2A). The enzyme families related to carbohydrate metabolism and cell wall degradation were characterized such as 229 glycosyl hydrolase (GH), 44 glycosyl transferase (GT), 51 carbohydrate esterase (CE), 94 carbohydrate-binding module (CBM), 67 auxiliary activity (AA), and 21 polysaccharide lyase (PL; Figures 2B–G). In the glycosyl hydrolase class, the most common CAZymes were GH16 and GH43. Twelve of the 229 GH families demonstrated the existence of three or more genes. The GH43 family had the most genes (28 genes) followed by GH16 (21), GH18 (19), and GH10 (7; Figure 2D). The secretome of *F. udum* also contained members of 10 CE families and CE5 (11 genes) had the most genes, followed by CE6 (10), CE3 (7), and CE4 (5; Figure 2F). Additionally, 4 PL families were anticipated that included PL1 (11 genes), PL3 (5), PL4 (3), and PL9 (2; Figure 2B). The other classes like AA (10 families), CBM (16), and GT (19) that play indirect roles in the degradation of carbohydrates were also predicted (Figures 2C,E,G). The CBM-13, AA7, and GT-4 families were found highly prevalent in the analyzed *F. udum* secretome.

### Mining of pathogenicity genes

**Figure 3:**
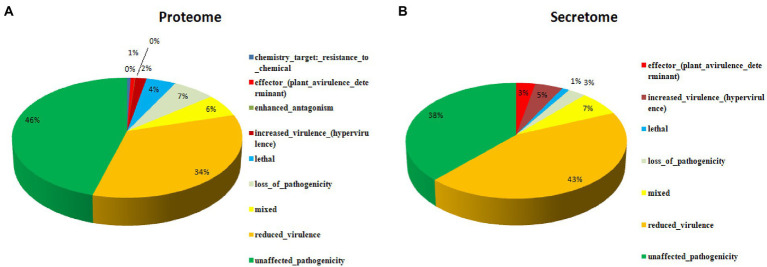
Putative pathogenicity-related genes associated with the secretome as annotated against the pathogen-host interaction database, **(A)** Proteome, and **(B)** Secretome.

A total of 16,179 predicted coding were sequences searched and validated against PHI database to identify virulence genes. As a result, 5,261 coding sequences were identified that are having active role in host-pathogen interaction. These proteins were further categorized into unaffected pathogenicity (46%), reduced virulence (34%), loss of pathogenicity (7%), mixed nature (6%), lethal (4%), increased virulence (2%), genes related to effectors (plant avirulence determinant; 1%), enhanced antagonism (~0%) and chemistry target resistance to chemicals (~0%; Figure 3A). In order to identify the putative pathogenicity-related genes, 1,439 secretory proteins were annotated against PHI database and 421 genes were identified (Supplementary Table S6). Furthermore, we confirmed that 183 genes were related to reduced virulence, 160 genes were unaffected by pathogenicity, 29 genes were of mixed nature, 13 genes related to effectors (plant avirulence determinant), 19 genes related to increased virulence, 12 genes related to loss of pathogenicity, and 5 genes were related to lethal activity (Figure 3B).

### Comparative analysis for effector genes

In the secretome of F-02845, a total of 7 effector genes that are encoding Xyloglucan-specific endo-beta-1,4-glucanase 1 (*XEG1*; 2 in no.), Cellulose-growth-specific protein (*MoCDIP4*; 3), 25 kDa protein elicitor-like protein (*PaNie 3*), avirulence gene/subtelomeric avirulence effector (*AVR-Pita*; 1), *MoCDIP3* (1), *MoCDIP1* (1), and Secreted virulence factor MC69 (*CoMC69*; 1) were identified. Further, we also compared these within the secretome of four different *Fusarium* spp., i.e., *F. oxysporum* f. sp. *lycopersici* (AAXH00000000), *F. graminearum* (AACM00000000), *F. proliferatum* (*FJOF00000000*), and *F. verticilliodes* (AAIM00000000). As a result, out of 7 effectors, only 5 (*XEG1*, *MoCDIP4*, *AVR-Pita*, *CoMC69*, and *MoCDIP1*) were common in all the *Fusarium* spp. with different copy numbers. PaNie and MoCDPI3 were not common in all the studied *Fusarium* spp. Interestingly, PaNie was not observed in *F. oxysporum* f. sp. *lycopersici* and *F. proliferatum.* Similarly, MoCDIP3 is absent in *F. graminearum* and *F. proliferatum.* The highest copy numbers of PaNie and MoCDIP3 were found in *F. udum* (3) and *F. verticilliodes* (3), respectively (Table 3).

**Table 3:** Effectors in secretome of different *Fusarium* species.

**Sr. No.**	**Protein Gene id**	**Gene-name**	***F. udum***	***F. lycopersici***	***F. graminarum***	***F. proliferatum***	***F. verticilliodes***
1.	G5A0G9	XEG1	2	2	1	2	1
2.	G4MVX4	MoCDIP4	4	2	4	6	5
3.	Q9SPD4	PaNie	3	0	1	0	1
4.	Q9C478	AVR-Pita	1	2	1	3	2
5.	H7CE70	CoMC69	1	1	1	1	2
6.	G4MX34	MoCDIP3	1	1	0	0	3
7.	G4N8Y3	MoCDIP1	1	1	1	1	1

### Characterization of repetitive elements

Repetitive elements in F-02845 genome were analyzed and found 895,132 bp (1.59%) of the total genome. A total of 128 transposable and 128 LTR were found in the genome. Among the LTRs, Tyl/copia (68) and gypsy/DIRS1 (60) represented more predominant in numbers (Table 4). The relative density and abundance of different SSRs were studied to get the genetic diversity of different SSRs in the F-02845 genome. A total of 4,921 SSRs representing 80,878 bp of the genome were identified. We observed relative density and abundance of 87.28 and 1434.41, respectively, for SSRs in the assembled genome. Details of different types of SSRs obtained from the whole genome of F-02845 are shown in Supplementary Table S7.

**Table 4:** Identified transposable elements in *F. udum* F-02845 genome showing highest count of Ty1/Copia.

**Sr. No.**	**Elements**	**Number of elements**	**Length occupied**	**Percentage of sequence**
	**Retroelements**	128	88,783 bp	0.16%
1.	SINEs	0	0 bp	0.00%
2.	Penelope	0	0 bp	0.00%
3.	LINEs:	0	0 bp	0.00%
4.	CRE/SLACS	0	0 bp	0.00%
5.	L2/CR1/Rex	0	0 bp	0.00%
6.	R1/LOA/Jockey	0	0 bp	0.00%
7.	R2/R4/NeSL	0	0 bp	0.00%
8.	RTE/Bov-B	0	0 bp	0.00%
9.	L1/CIN4	0	0 bp	0.00%
	**LTR elements**	128	88,783 bp	0.16%
1.	BEL/Pao	0	0 bp	0.00%
2.	Ty1/Copia	68	29,240 bp	0.05%
3.	Gypsy/DIRS1	60	59,543 bp	0.11%
4.	Retroviral	0	0 bp	0.00%
	**DNA transposons**	128	61,613 bp	0.11%
1.	hobo-Activator	5	703 bp	0.00%
2.	Tc1-IS630-Pogo	22	14,498 bp	0.03%
3.	En-Spm	0	0 bp	0.00%
4.	MuDR-IS905	0	0 bp	0.00%
5.	PiggyBac	14	3,987 bp	0.01%
6.	Tourist/Harbinger	0	0 bp	0.00%
7.	Other (Mirage, P-element, Transib)	0	0 bp	0.00%
8.	Rolling-circles	0	0 bp	0.00%
9.	Unclassified:	3	423 bp	0.00%
	**Total interspersed repeats**		150,819 bp	0.27%
1.	Small RNA:	50	16,939 bp	0.03%
2.	Satellites:	0	0 bp	0.00%
3.	Simple repeats:	13,725	636,194	1.13%
4.	Low complexity:	1,776	91,188 bp	0.16%

### Comparison of orthologous genes

**Figure 4:**
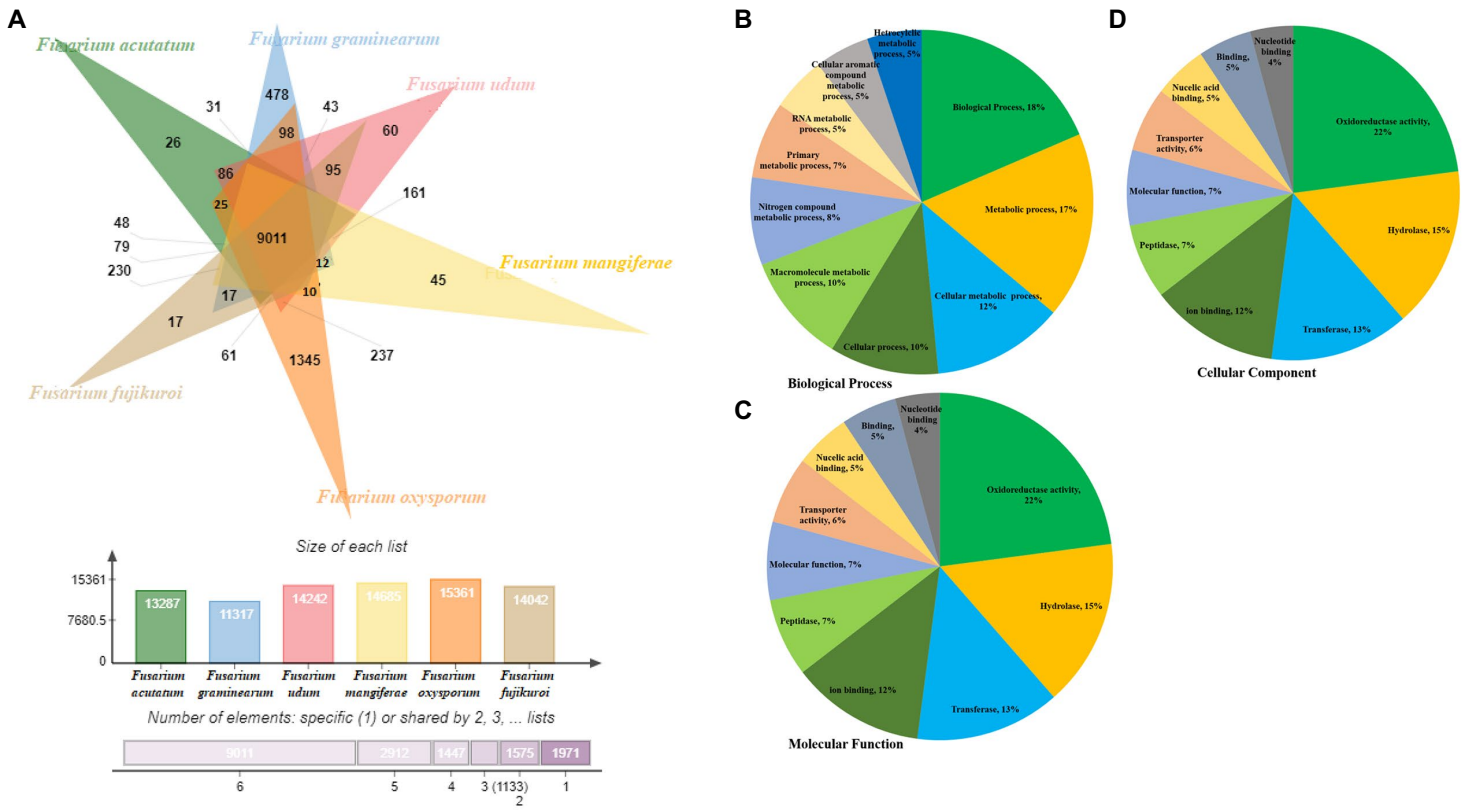
Comparison of orthologous genes between different ascomycetes fungus genome. **(A)** Functional annotation of the shared clusters showing top hits of different category, **(B)** Biological process, **(C)** Molecular function and **(D)** Cellular component.

Comparative genome analysis of predicted proteome of F-02845 was performed with six other species of *Fusarium* (Figure 4A). The genome statistics (size, GC%, accession number) of these genomes have been mentioned in Supplementary Table S8. Results indicated that these species formed 18,274 clusters, out of which the *F. udum F-02845* shared a total of 14,242 clusters, 9,011 common clusters, and 60 singletons. The F-02845 has shared a total of 237 common clusters with other *Fusarium* species; 161 clusters with *F. mangiferae;* 95 common clusters with *F. Fujikuroi,* 86 common clusters with *F. acutatum*; and 43 common clusters with *F. graminearum*. Further, functional annotation was done for these common clusters and a total of 202 GO terms were assigned to BP, which included biological processes (18%), metabolic processes (17%), cellular metabolic processes (12%), cellular processes (10%), macromolecular metabolic processes (10%), etc. (Figure 4B). Similarly, 46 GO terms were assigned for MF, which includes oxidoreductase activity (22%), hydrolase (15%), transferase (13%), ion binding (12%), peptidase (7%), molecular function (7%) etc. (Figure 4C). Besides this, 25 GO terms were assigned for the CC, which include membrane functions (21%), cellular component (16%), cell part (16%), nucleus (10%), intracellular (10%), mitochondria (7%) etc. (Figure 4D).

### Wet lab validation of few effector genes

Among 14 SSR primers designed for a few effector genes (*SIX* genes), the primer set SIX1A3 (forward primer GCCCAGGTCGTAAATAGTGAGA and reverse primer GCAGACTCAACTCCAAATAGGC) was validated through PCR. The amplification of SIX1A3 gene with desired amplicon size (300 bp) was found in all seven strains of *F. udum* (F-02842, F-02843, F-02844, F-02845, F-02848, F-02850, and F02851).

## Discussion

Pigeon pea is a herbaceous pulse crop, predominantly cultivated in tropical and subtropical climates and is vulnerable to more than hundreds of pathogens (Nene et al., 1981). Among the diseases, the major fungus affecting pigeon pea is *F. udum* is the major concern. Since, chemical and biological management of this disease has witnessed limited success against *F. udum* wilt. The whole genome sequencing and analysis of virulence-related genes could bring a better understanding of wilt disease for devising appropriate management strategies. Therefore through this study, we analyzed the first draft genome sequence of *F. udum* F-02845 for virulence related genes.

The assembled genome of F-02845 was retrieved, which constituted size of 44.62 Mb with 48.3% of G + C content and 42,598 bp N_50_ length. It is worth to mention here that, our assembled genome was near to complete and more accurate than recently reported genome of NRL 25194. In comparison to NRL 25194 strain, we found F-02845 harbors higher physiological complexity owing to its larger genome size. The larger genome size could be particularly associated with the larger biosynthetic machinery. However, this difference could not be established with respect to the virulence of the pathogen. Similarly, Dobbs et al. (2020) through whole genome sequencing of two strains of pathogen causing koa wilt (*Fusarium oxysporum* f. sp. *koae*) reported differences in genome sizes of non-pathogenic strain Fo170 (50 Mb) as compared to pathogenic strain Fo44 (48 Mb).

Annotation of F-02845 genome revealed the presence of genes related to cell physiology and functioning. The KOG annotation showed the presence of 1,060 putative pathogenicity genes along with other genes for cellular processes. These pathogenicity genes are of great importance when looking at the economic loss caused by the pathogen. To see early occasions in plant-pathogen (*F. udum*) interactions, it was important to investigate the pathogen secretome to recognize secreted proteins that help to organize pathogenicity. Usually, a subset of the secretome is composed of proteins whose presence is needed to initiate infection and their expulsion from the secretome would bring about pathogens with diminished or no virulence (Ranganathan and Garg, 2009). In this study, a total number of 14,673 and 16,179 protein-coding genes with an average length of 1,365 bp were predicted. These genes were further functionally annotated using BlastP and were applied to the PHI database in order to search for the virulence genes present in the fungal genome. F-02845 genome identified to contain 1,439 classical secretory proteins with successfully annotated function for 1,305 secretory proteins. It is a well-established fact that for successful invasion, pathogen secretes enzymes to destroy the plant cell walls. In addition, enzymes related to carbohydrate metabolism determine the efficacy of the pathogens to grow and their aggressiveness to cause disease. CAZymes play an important role in degrading plant biomass and have many associated families like carbohydrate esterases, glycosyl hydrolases, and polysaccharide lyases that are involved in cell wall degradation (Ospina-Giraldo et al., 2010; Zhao et al., 2013; Barrett et al., 2020). In this study, we identified 506 secretory proteins (CaZymes) with cell wall degradation functions. The presence of these secreted enzymes could contribute to active entry of pathogens into the host cells. In particular, the host-specific populations of pathogens had various enzymes which share comparative functions. For instance, the pigeon pea wilt strains share six CAZyme copies associated with the hydrolysis of different cell wall components such as chitin, pectin, and rhamnose, along with the breakdown of glucose, xylan, and mannose (Clerivet et al., 2000). This suggests strong enzymatic capability for biomass degradation. Cell-wall breakdown discharges pectins into the xylem vessels, which could unexpectedly act as a barrier to additional microbe growth. At this moment pectin-degrading enzymes play crucial role in pathogenesis as they remove pectin barriers and help the pathogen to spread across the plant tissues and cause disease symptoms. The ability to utilize plant carbon is indicative of *F. udum*’s ability to survive inside plants before the appearance of disease symptoms (Soanes et al., 2008), which is also evident from the field infection of *F. udum*.

PHI database analysis of the secretome revealed 160 genes associated with pathogenicity loss, 29 genes of mixed nature, 19 genes associated with increased virulence, 13 genes associated with effectors (plant avirulence determinant), and 5 genes associated with lethality. Targeting these genes would open a new arena in biocontrol of this pathogen. In the F-02845 secretome, 13 genes related to effectors *viz.*, XEG1, MoCDIP4, PaNie, AVR-Pita, MoCDIP3, MoCDIP1 and CoMC69 were identified. Effectors are avirulence proteins or products involved in pathogenicity that have the ability to manipulate host cell structure and function and thereby facilitates infection. They often contribute quantitatively to pathogen aggressiveness and are dispensable for the pathogen life cycle. These effectors also play an important role in taking up of nutrients from the host tissues or pathogens self-defense (Rovenich et al., 2014; Fatima and Senthil, 2015). These could be possibly one of the key virulence determinants, which provide ecological fitness to the *F. udum*. Djamei and Kahmann (2012) analyzed the genome of biotrophic maize pathogen *Ustilago maydis* and predicted 550 secreted proteins which were upregulated during host colonization. They also reported that the *U. maydis* secretes core and organ-specific effectors. The main roles of core effectors are that they suppress plant defense during the penetration stage and organ-specific effectors infect different plant tissues (Skibbe et al., 2010; Djamei and Kahmann, 2012). The presence of core effectors could be helpful for *F. udum* to bypass the host defense system and colonize host tissues. It has been widely reported that suppressing host defense is useful in establishing disease. Effector-encoding gene clusters were found in the *U. maydis* genome. The largest effector gene cluster, 19A, contains 23 genes. They are differentially induced when different plant organs are colonized. It has been noticed that removal of the complete 19A cluster terminates tumor formation in maize plants, whereas deletion of individual genes shows a minor reduction in virulence (Kamper et al., 2006; Brefort et al., 2014). Therefore, it would be possible that controlling these *F. udum* effector encoding clusters could significantly reduce the disease symptoms.

*Fusarium* has a number of different *formae speciales*, and each of them is harmful to a different host plants. The diversity of well-known effector gene *SIX* is demonstrated to be strongly up-regulated during colonization of the host plant by *F. oxysporum* f. sp. *lycopersici* (Houterman et al., 2007). More recently, eight tiny secreted fungal proteins known as SIX1 to SIX8 were discovered in the xylem sap of infected plants. These effector genes are also known to play an important role in virulence as these genes are absent in non-pathogenic *F. oxysporum* (van Dam et al., 2016). The effector SIX3 (AVR2) is established in the cell of the host and incorporated with SIX5 activates resistance and cell death in tomato plants carrying the I-2 gene (Houterman et al., 2009). SIX1 (AVR3) induced resistance in plants containing the I-3 gene from the wild tomato *Solanum pennellii* (Catanzariti et al., 2015). On parallel lines, two copies of XEG1 (Xyloglucan-specific endo-beta-1,4-glucanase 1) were also noticed in the *F. udum* genome. This observation provides an important clue regarding the probable role of this enzyme in cell wall degradation and pathogen invasion, as documented by earlier workers (Bourquin et al., 2002; Baumann et al., 2007).

In the comparison study of effectors among different *Fusarium* species, we found that out of seven secretory effectors, five were common to other *Fusarium* species and two were specific to certain species only. This could be one of the plausible reasons behind the specific pathogenicity behavior of *F. udum*. The higher copy numbers of *MoCDIP4* and *PaNie* genes are identified in the *F. udum* genome. The possible connection could be with the pathogenic establishment of *F. udum* in the host plant as MoCDIP1-MOCDIP5 secretory effectors are responsible for the host cell death (Chen et al., 2013). It may be contributing to the silencing of the host defense response. Cell death–inducing proteins of *Magnaporthe oryzae* have been reported as cell death–inducing proteins and cause necrosis in both monocots and dicots (Chen et al., 2013). They are expressed at the late infection stage of the host. Similarly, a novel protein elicitor (PaNie234) isolated from the pathogenic oomycete *Pythium aphanidermatum* activated the programmed cell death and *de novo* formation of 4-hydroxybenzoic acid in cultured cells of *Daucus carota* (Veit et al., 2001). Recently, a fungal effector protein was found to suppress the host plant’s polygalacturonases-inhibiting proteins (PGIP), which inhibit fungal polygalacturonases (PG). The PG is secreted by pathogens to degrade host cell walls (Wei et al., 2022). The presence of MoCDIP4 and PaNie effectors in the *F. udum* genome indicated another mechanism of the pathogen in breaching the host’s physical defense.

Apart from secretory effector proteins, comparative genome analysis suggested variations in the number of total proteins in the genomes of different *Fusarium* spp. For instance, total proteins reported in *F. udum F-02845, Fusarium acutatum* (*JAADJF000000000*), *Fusarium graminearum PH 1*, *Fusarium mangiferae* (*FCQH00000000*), *Fusarium oxysporum 2,478, Fusarium poae FPOA1,* and *Fusarium_fujikuroi IMI 58289* genomes were 16,179, 14,081, 13,313, 15,804, 18,769, 14,740, and 15,371, respectively. These species formed 18,274 clusters, out of which F-02845 shared a total of 14,242 clusters, 9,011 common clusters, and 60 singletons. Comparative genome analysis of closely related species is the best perspective for the recognition of virulence determinants (Kamper et al., 2006). Sharing common clusters among other *Fusarium* spp. suggests that *F. udum* has common pathogenicity determinants, which could be targeted for common control strategy for all related pathogens.

Repetitive DNA plays an important role in the evolution of eukaryotic genomes. It causes genetic and beneficial changes in the evolution of pathogens (Castanera et al., 2016). In the present study, we identified 128 transposable elements. In addition to this, repetitive sequences related to epigenetic control of the expression of effector genes as part of the coordinated infection strategy (Razali et al., 2019). Simple sequence repeats (SSRs) are the repetitive DNA tracks distributed throughout the genome. These play a vital role in deciphering the genetic diversity among different fungal genera, species, and strains (Kumar et al., 2012, 2013; Singh et al., 2014; Rai et al., 2016; Kashyap et al., 2020). Here we report, identification of total 4,921 SSR with a length of 80,878 bp in the F-02845 genome, which could be instrumental in deciphering evolutionary relatedness (Rao et al., 2018; Kashyap et al., 2019).

Some of the genes and gene clusters identified in the present study could play a crucial role in pathogenesis and host-pathogen interaction for wilt disease development in pigeon pea. Such virulence genes could be used for functional characterization to recognize the infection mechanism of *F. udum* causing the wilt of pigeon pea. Establishing the molecular basis of host infection could be further utilized in marker assisted selection, CRISPR-Cas9 based genome editing, and other molecular approaches for developing disease resistance in crop plants and effective management of disease in the farmer’s field.

## Conclusion

Through this study, we report whole genome and secretome analysis of most virulent strain of *F. udum* F-02845, causing pigeon pea wilt. Understanding of the genes responsible for virulence and secondary metabolite production in fungus will help to explain the mechanisms for virulence functional in fungus and to develop novel strategies for disease management of *Fusarium* wilt. Various genes and their secreted proteins identified in this study, which are crucial for disease development, could be of greater significance in deciphering pathogenic determinants of *F. udum*. Comparison of orthologous genes in this study from other similar pathogen genomes resulted in identification of common set of genes, which could be used to explain the behavior of *F. udum* in respect to disease development. The information generated from this study has not only helped in deciphering virulence determinants, but also helpful in understanding the pathogen’s complex ecological behavior that has yet to be discovered. This would add to the plant health promotion efforts in sustainable agriculture.

## Data availability statement

The datasets presented in this study can be found in online repositories. The names of the repository/repositories and accession number(s) can be found at: NCBI GenBank – NIFK00000000; NCBI Sequence Read Archive – SRP157084.

## Author contributions

AlS and PK conceived the idea and designed the experiments. RS, JY, ASi, PT, AnS, and PK performed the experiments. AlS, RS, PS, and SS analyzed the data. AlS, RS, PS, and ASi wrote and edited the manuscript. All authors contributed to the article and approved the submitted version.

## Funding

This research was supported by the funds received from CRP-Genomics, Indian Council of Agricultural Research, New Delhi (India).

## Conflict of interest

The authors declare that the research was conducted in the absence of any commercial or financial relationships that could be construed as a potential conflict of interest.

## Publisher’s note

All claims expressed in this article are solely those of the authors and do not necessarily represent those of their affiliated organizations, or those of the publisher, the editors and the reviewers. Any product that may be evaluated in this article, or claim that may be made by its manufacturer, is not guaranteed or endorsed by the publisher.

## Footnotes

^1^ http://pgrc.ipkgatersleben.de/misa

^2^ http://wsmartins.net/websat/

^3^ http://bioin.f.uni-greifswald.de/augustus

^4^ http://www.cbs.dtu.dk/services/SignaLP/
