# Supplementary material for: Virulence and pathogenicity determinants in whole genome sequence of Fusarium udum causing wilt of pigeon pea
Source: Front Microbiol. 2023 Feb 17;14:1066096. doi: 10.3389/fmicb.2023.1066096 (PMC9981795; doi:10.3389/fmicb.2023.1066096)
Supplement: Supplementary file 1 [file Data_Sheet_1.docx]

**Supplementary data**

**Table S1: Pot experiment for pathogenicity analysis of *Fusarium udum* isolates**

| **Treatments** | **Mortality (%) in four different pigeon pea cultivars** | | | |
| --- | --- | --- | --- | --- |
|  | **BAHAR** | **BRG2** | **DA 11** | **MAL 13** |
| Uninoculated control | 0.00^e^ | 0.00^d^ | 0.00^e^ | 0.00^e^ |
| F-02842 | 50.00^d^ | 43.33^b^ | 36.67^d^ | 46.67^cd^ |
| F-02843 | 63.33^c^ | 50.00^b^ | 53.33^c^ | 50.00^c^ |
| F-02844 | 46.67^d^ | 20.00^c^ | 30.00^d^ | 36.67^d^ |
| F-02845 | 96.67^a^ | 70.00^a^ | 83.33^a^ | 73.33^a^ |
| F-02848 | 80.00^b^ | 66.67^a^ | 70.00^b^ | 63.33^ab^ |
| F-02850 | 70.00^bc^ | 40.00^b^ | 56.67^c^ | 53.33^bc^ |
| F-02851 | 80.00^b^ | 50.00^b^ | 63.33^bc^ | 66.67^a^ |
| SED | 5.94 | 5.16 | 5.67 | 4.88 |
| CD | 12.75 | 11.06 | 12.16 | 10.47 |

Means within columns with different letters are significantly different as determined by LSD (P < 0.05), results in bold indicate treatments with significantly highest mortality

**Table S2:** Genome assembly statistics of *Fusarium udum* F-02845 genome

| **Sr. No.** | **Strain Id** | **F-02845** |
| --- | --- | --- |
|  | Total number of scaffolds | 712 |
|  | Total number of bases(bp) | 56,381,318(~56.38Mb) |
|  | Minimum scaffold length (bp) | 501 |
|  | Maximum scaffold length (bp) | 6,511,407 (~6.5Mb) |
|  | Average scaffold length (bp) | 79187.24 (~0.8Mb) |
|  | N50 Scaffold size (bp) | 5,402,168 (~5 Mb) |
|  | (G+C)% | 42.44% |
|  | #A | 15994469 (28.37%) |
|  | #T | 16028052 (28.43%) |
|  | #C | 11956531 (21.21%) |
|  | #G | 11973238 (21.24%) |
|  | #N’s | 429028 (0.76%) |

**Table S3:** Protein-coding genes of *Fusarium udum* (attached as MS excel sheet)

|  | ***F. udum* F-02845** | ***F.* udum NRL 25194** |
| --- | --- | --- |
| **Genome size (Mb)** | 56.38 | 44.61 |
| **Scaffold** | 712 | 2,353 |
| **Contigs** | 21,770 | 2,389 |
| **N50** | 4952 | 42,598 |
| **L50** | 3,333 | 307 |
| **GC%** | 42.4 | 48.3 |
| **No of genes predicted** | 16,179 | 21,062 |

**Table S4:** Comparison between the two reported whole genome of the *Fusarium udum*

**Table S5:** Secretory proteins of *Fusarium udum* (attached as MS excel sheet)

**Table S6:** Secretory proteins of *Fusarium udum* annotated against the pathogen-host interaction database (PHI database) (attached as MS excel sheet)

**Table S7:** Number of SSRs identified in the *F. udum* isolate F-02845 genome and their distribution to different repeat type classes

| **Sr. No.** | **Unit size** | **No of SSR** |
| --- | --- | --- |
|  | 1 (1/10) | 1957 |
|  | 2 (2/6) | 1543 |
|  | 3 (3/5) | 1051 |
|  | 4 (4/5) | 173 |
|  | 5 (5/5 | 125 |
|  | 6 (6/5) | 72 |
|  | Total | 4921 |

**Table S8:** The genome statistics of different strains of *Fusarium* used for comparison of orthologous genes

| **Particulars** | ***F. udum* F02845** | ***F. acutatum*** | ***F. graminearum*** | ***F. mangiferae*** | ***F. oxysporum* 4287** | ***F. fujikuroi* IMI 58289** |
| --- | --- | --- | --- | --- | --- | --- |
| Genome size (Mb) | 56.38 | 48.3 | 43.33 | 46.29 | 53.91 | 43.8323 |
| Scaffold | 712 | 2,353 | 982 | 254 | 499 | 12 |
| Contigs | 21,770 | 2,389 | 1,042 | 514 | 504 | 65 |
| N50 | 4952 | 42,598 | 114,620 | 629,886 | 1,338,693 | 1,182,607 |
| L50 | 3,333 | 307 | 111 | 23 | 11 | 13 |
| GC% | 42.4 | 48.3 | 48.5 | 48.3 | 47.68 | 47.46 |


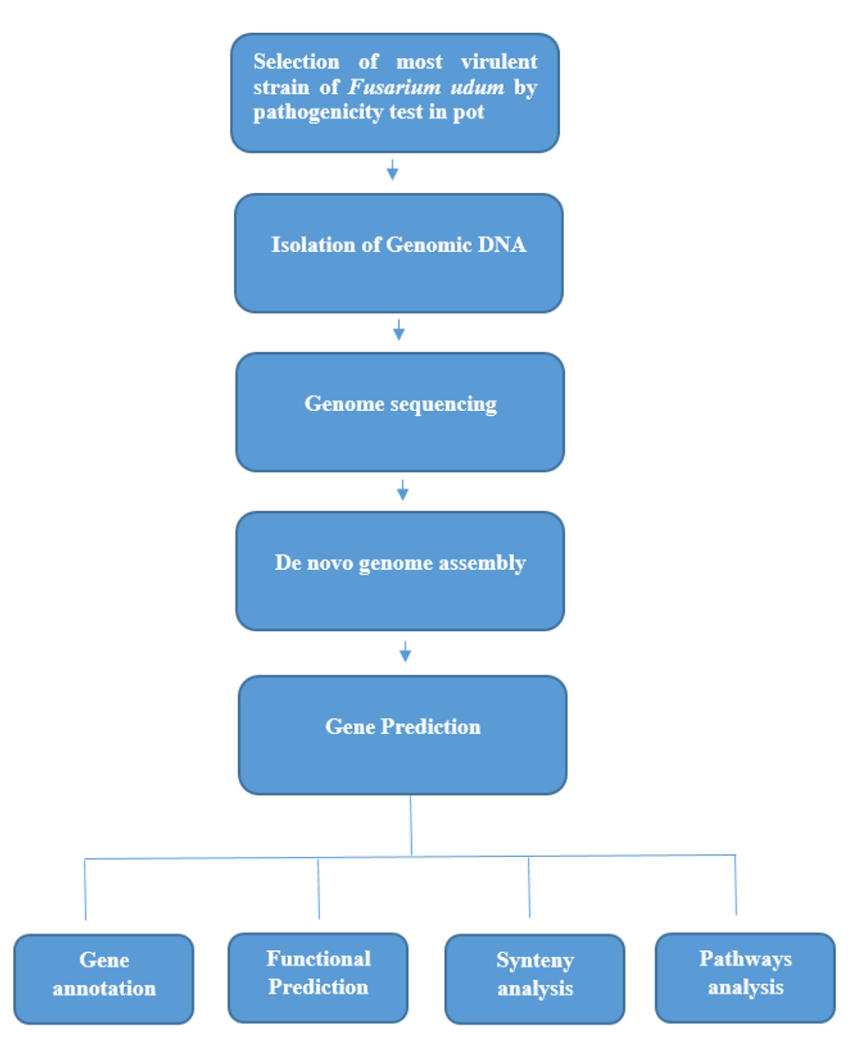


**Figure S4:** Workflow of data analysis

**
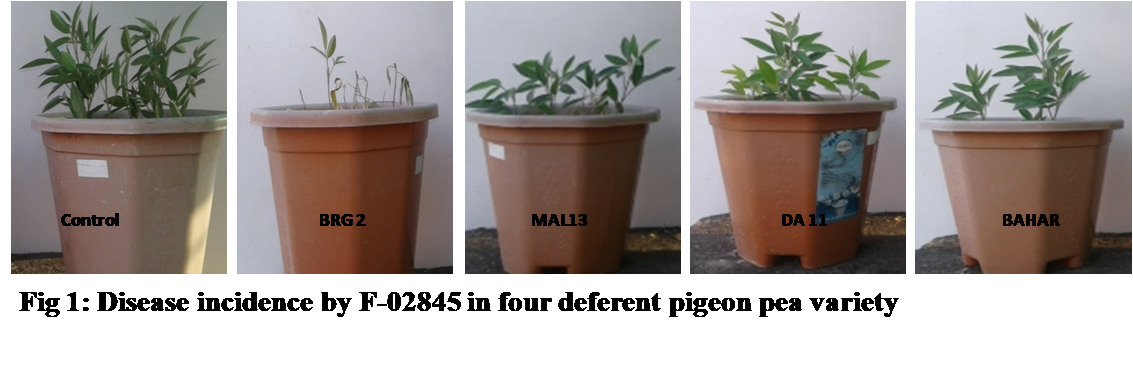
**

**Figure S2:** Disease incidence by *F. udum* F-02845 in pigeon pea variety BRG2

**
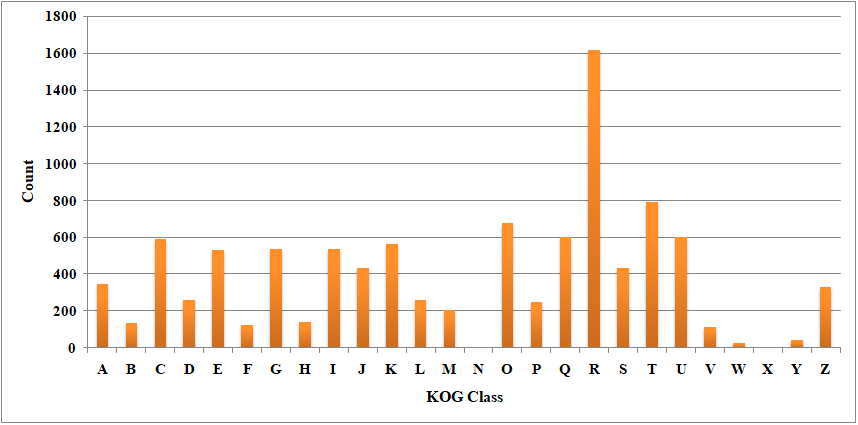
**

**Figure S3:** Functional annotation of *F. udum* genome using KOG database

**
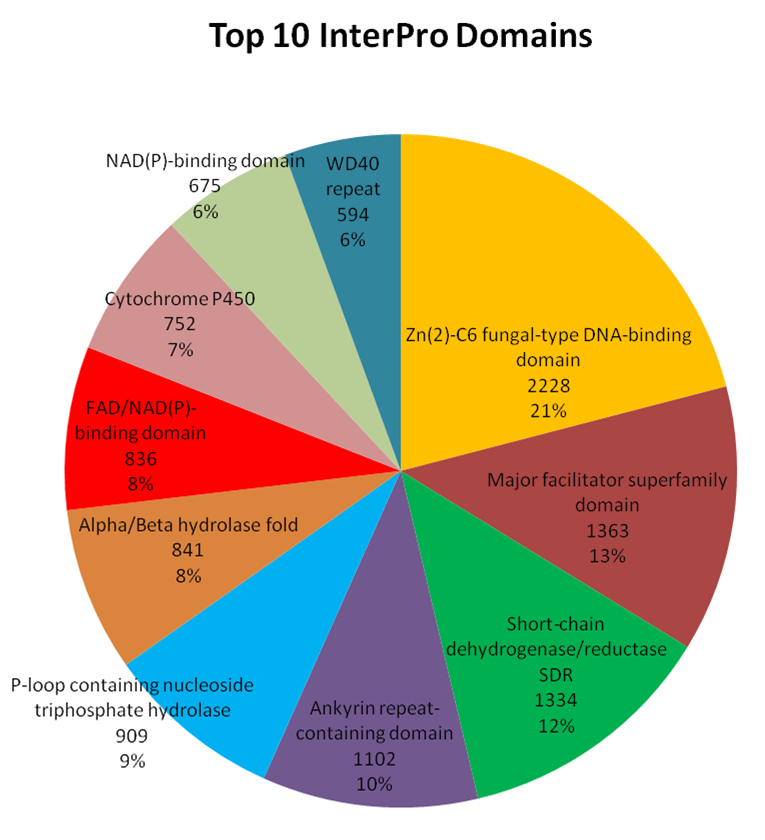
**

**Figure S4**: The domain analysis of *F. udum* genome using InterproScan-V5 showing unique Interpro domains


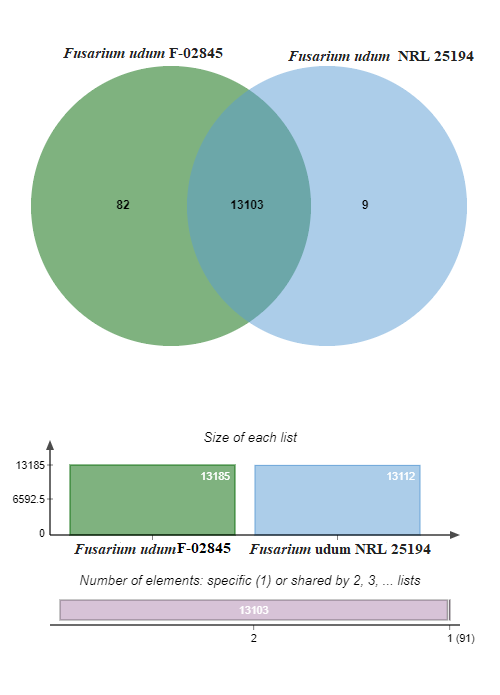


**Figure S5**: Comparison between the two reported whole genomes of the *Fusarium udum*


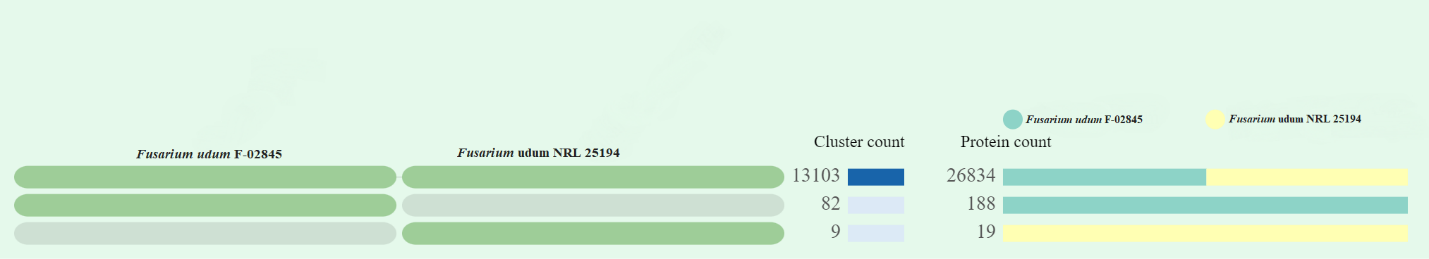


**Figure S6**: Overlap between the two reported whole genomes of the *Fusarium udum*
